# Feasibility of joystick guided colonoscopy

**DOI:** 10.1007/s11701-015-0511-6

**Published:** 2015-05-10

**Authors:** Esther D. Rozeboom, Ivo A. M. J. Broeders, Paul Fockens

**Affiliations:** Department of Robotics and Mechatronics, Faculty of Electrical Engineering, University of Twente, P.O. Box 217, 7500 AE Enschede, The Netherlands; Department of Robotics and Mechatronics, University of Twente, Enschede, The Netherlands; Department of Surgery, Meander Medical Center, Amersfoort, The Netherlands; Department of Gastroenterology and Hepatology, Academic Medical Centre, University of Amsterdam, Amsterdam, The Netherlands

**Keywords:** Flexible endoscopy, Colonoscopy, Joystick, Feasibility

## Abstract

The flexible endoscope is increasingly used to perform minimal invasive interventions. A novel add-on platform allows single-person control of both endoscope and instrument at the site of intervention. The setup changes the current routine of handling the endoscope. This study aims to determine if the platform allows effective and efficient manipulation to position the endoscope at potential intervention sites throughout the bowel. Five experts in flexible endoscopy first performed three colonoscopies on a computer simulator using the conventional angulation wheels. Next they trained with the joystick interface to achieve their personal level of intubation time with low pain score. 14 PhD students (novices) without hands-on experience performed the same colonoscopy case using either the conventional angulation wheels or joystick interface. Both novice groups trained to gain the average expert level. The cecal intubation time, pain score and visualization performance (% of bowel wall) were recorded. All experts reached their personal intubation time in 6 ± 6 sessions. Three experts completed their learning curve with low pain score in 8 ± 6 sessions. The novices required 11 ± 6 sessions using conventional angulation wheels, and 12 ± 6 sessions using the joystick interface. There was no difference in the visualization performance between the novice and between the expert groups. This study shows that the add-on platform enables endoscope manipulation required to perform colonoscopy. Experts need only a relatively short training period. Novices are as effective and as efficient in endoscope manipulation when comparing the add-on platform with conventional endoscope control.

## Introduction

The flexible endoscope is increasingly used to perform minimal invasive interventions in the gastrointestinal tract. Up to 40 % of screening colonoscopies require the removal of at least one polyp from the large bowel [1, 2]. Also large defects can be removed endoscopically using complex procedures such as endoscopic mucosal resection and submucosal dissection [3–6]. Four hands are required to control the endoscope and its instrument. The endoscopist needs to master a combination of accurate tip angulation, shaft management and instrument insertion, while communicating with the endoscopic assistant to actuate the instrument and hold the endoscopic shaft when needed [7–11].

Several innovative endoscopes have been developed to reduce the effort of endoscope steering [12–14]. These redesigned endoscopes require a substantial investment in purchase of materials and training. We developed an add-on platform that allows single-person control of a conventional endoscope and instrument at the intervention site [15].

Previous studies showed that the add-on platform with joystick interface increases efficiency of endoscope tip positioning compared to the conventional angulation wheels [16, 17]. Additionally, single-person control of an endoscope and its instrument increases efficiency and satisfaction in a pick-and-place task [15].

**Fig. 1:**
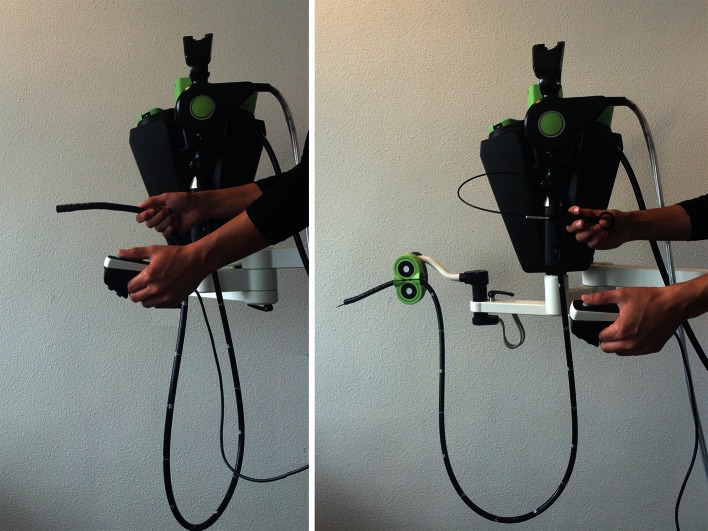
Add-on platform with joystick interface. The endoscopist maneuvers the endoscope to the intervention site with the endoscope already docked to the add-on platform (*left*). After reaching the intervention site, the shaft is held in position by the easy click-on system, freeing the right hand to manipulate an instrument (*right*)

The next step is to verify if endoscopists can reach the intervention site without the interruption of docking the add-on platform. Ideally, the endoscopist introduces the endoscope to the site of interest with the endoscope already docked to the add-on platform. At the intervention site, the endoscopist clicks the shaft in a holding system [18] (Fig. 1). This releases the right hand to position and actuate an instrument. The left hand continuously controls the endoscopic tip position with a remote intuitive interface such as a joystick. Small shaft position corrections can be applied using the same remote interface. This study aims to verify if endoscopists can reach the intervention site using the add-on platform. Endoscopists should be able to position the endoscope to potential intervention sites throughout the gastrointestinal tract.

The add-on platform changes the current routine of endoscope manipulation. In the conventional setup, torquing of the rotation stiff endoscopic shaft is the result of a combined effort by the left shoulder, wrist and right hand. Using the platform, the user holds the remote interface in his left hand. Scope rotation now depends entirely on the right hand. Shaft manipulation is critical for adequate endoscopy, with colon loop management being the most difficult challenge [7].

The aim of this study is to verify if our add-on platform with joystick interface enables adequate endoscope manipulation to position the endoscope throughout the bowel. To evaluate the potential of this module, a learning curve is recorded for both experts and novices.

## Methods

### Participants

Two groups of participants were involved; experts in gastrointestinal endoscopy and novices. The expert group consisted of five practicing endoscopists who had completed between 500 and >5000 colonoscopies in their careers. There were two male and three female experts with a median age of 46 ± 7 years. All experts were right-handed.

The novice group consisted of fourteen PhD students from the department of Gastroenterology and Hepatology of the Academic Medical Center, Amsterdam, who were in their second to fourth year. The novices had no prior experience in steering a flexible endoscope. They were divided in two groups, conventional or add-on platform with joystick interface (hereupon referred to as ‘joystick’ group). Each group consisted of three men and four women, with a median age of 28 ± 2 years. There were two left handed participants in the conventional group.

None of the experts or novices had previous experience with endoscope manipulations using the platform with joystick interface.

### Simulator

All sessions were carried out on case 6 of the Introduction to Colonoscopy module of the AccuTouch virtual reality endoscopy simulator (CAE Healthcare, Montreal, Quebec, Canada; previously Immersion Medical, Gaithersburg, MD, USA). The system consists of real-time computer graphics, an interface device with force-feedback on the endoscope shaft and audible response indicating patient discomfort. Case 6 is the most difficult case in this version of the simulator, with maximal loop formation and pain scores. This case requires a high level of adequate tip steering and shaft manipulation to complete cecal intubation with low pain score.

### Add-on platform

**Fig. 2:**
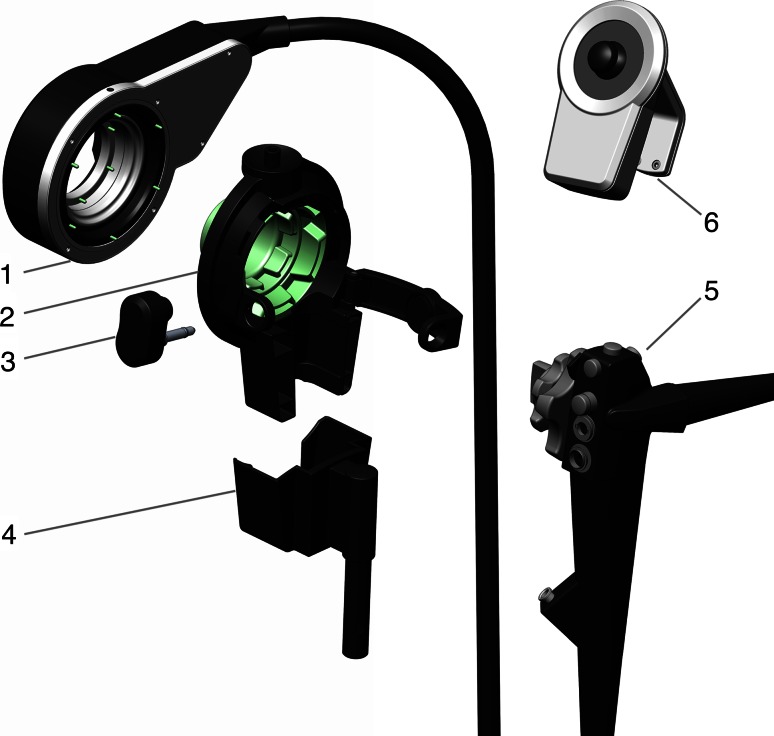
Exploded view of the add-on platform: *1* remote drive unit, *2* connection module, *3* plug, *4* docking station, *5* endoscope and *6* joystick controller

The add-on platform, described by Ruiter et al. [16], is designed to connect to a conventional endoscope. It consists of a stationary motor unit, which actuates the angulation wheels of the endoscope through a remote drive unit. The drive unit is connected to the angulation wheels through a connection module, fixed with a plug and placed in a docking station (Fig. 2).

**Fig. 3:**
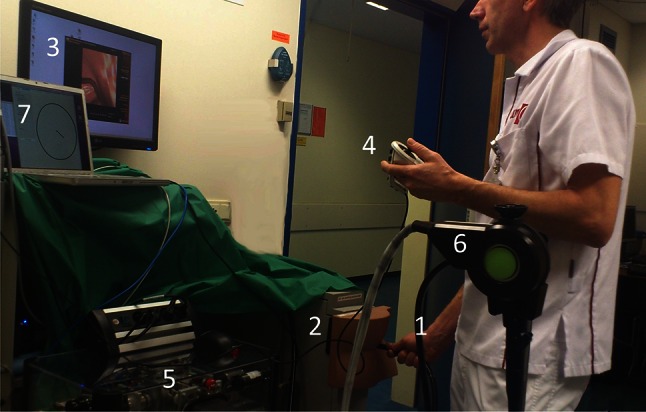
The test setup includes *1* a dedicated colonoscope, *2* simulator interface and *3* real-time computer graphics. The platform control consists of *4* a remote joystick, *5* stationary motor unit, *6* remote drive unit connected to the endoscope console and positioned in the docking station, and *7* an endoscope tip bending diagram

The user only holds the remote joystick in his left hand to control endoscope tip angulation, air/water and suction functions (Fig. 3). The right hand controls endoscope shaft introduction, rotation and withdrawal, similarly to the conventional steering method. A visual tip bending diagram informs the user of the tip’s angulation position and the steering direction necessary to straighten the tip (Fig. 3, nr 7).

### Procedure

All experts first performed the colonoscopy case three times using the traditional angulation wheels. Next, they practiced the same case using the platform with joystick interface until they reached a personal endpoint in their learning curve. The endpoint for the experts consisted of the average + one standard deviation of their conventional cecal intubation time (IT) and no severe or extreme pain (NP).

The novices were divided into two groups: one group used the conventional angulation wheels, and the other group used the setup with joystick interface. The end-IT for both novice groups was the average plus one standard deviation of the intubation time of the experts using the conventional angulation wheels. Novices also practiced to reach their end-IT with NP.

The NP endpoint was selected to enforce realistic endoscopic techniques like loop detection and straightening techniques. Without the NP endpoint, users are able to forcefully insert the scope into the simulator, leading to unrealistic outcomes.

Before the first session, all participants received written trial instructions including which parameters were recorded and the simulators cues to detect looping, successful straightening, the level of patient pain and how to recover lumen vision from a red out. They were also allowed to train 5 min on the first (easiest) colonoscopy case to gain familiarity with the simulator. During the sessions, participants were not allowed to use the simulator’s options for a virtual attending physician and external view of the endoscope. The sessions lasted 1–2 h on each occasion. Sessions included 5–10 min resting breaks, they could be repeated several times per week and continued over 2–7 weeks.

Evaluation parameters measured by the computer simulator were the IT, pain score (% of procedure time), bowel wall visualization (% of bowel wall) and withdrawal time. To enable comparison of visualization performance, participants were instructed to include a 6 min withdrawal time. This is the recommended clinical practice [19]. Afterwards, users were requested to select their preferred steering method.

### Statistical analysis

Statistical analysis was performed using IBM SPSS Statistics version 21. Differences between novices using conventional or joystick platform were analyzed using the Mann–Whitney test. Differences between experts using conventional or joystick platform were analyzed using Wilcoxon’s Matched Pairs test. For all tests, *P* values under 0.05 were considered statistically significant. Values are expressed as the mean (±standard deviation).

## Results

**Fig. 4:**
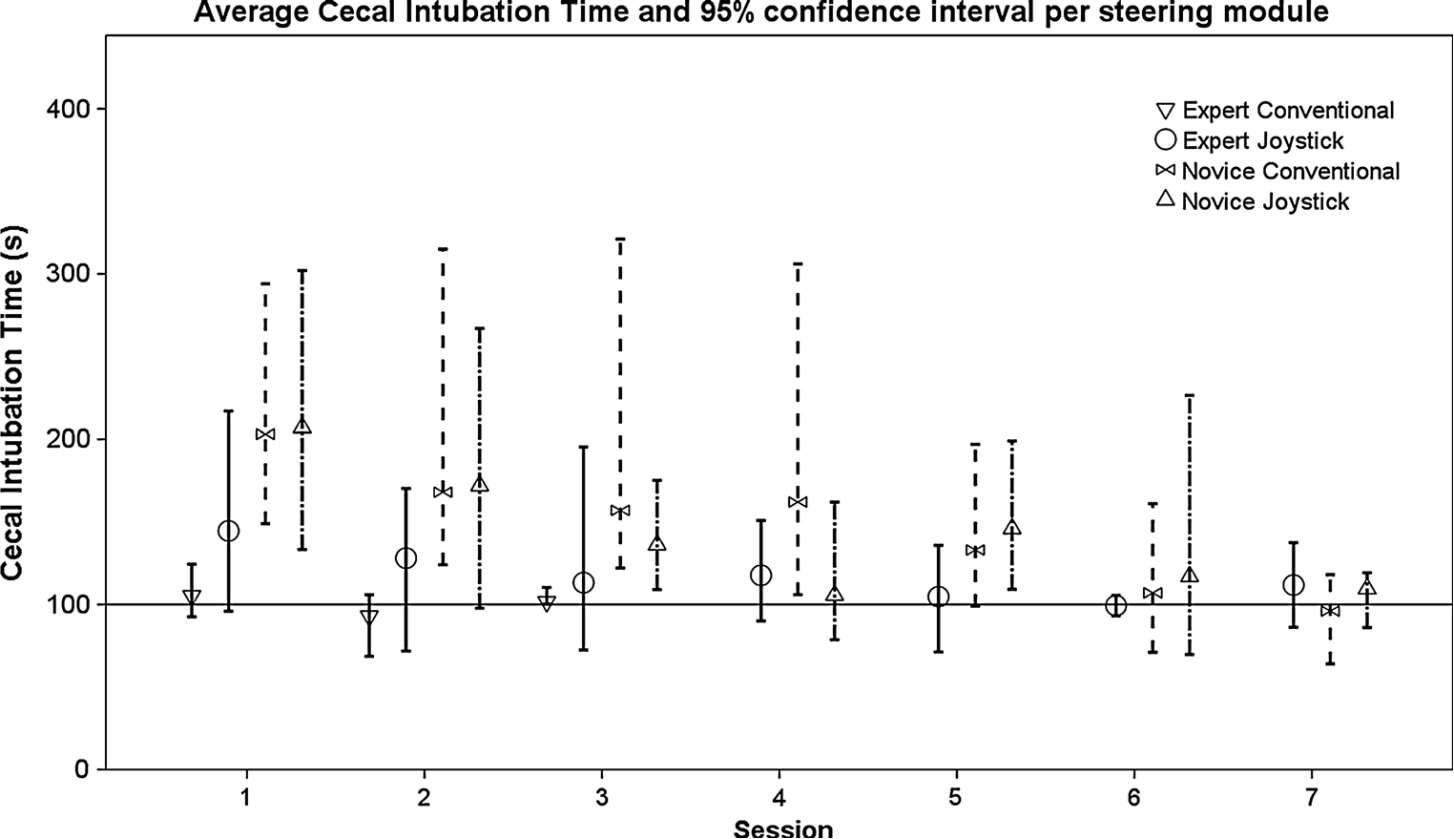
Learning curves of the cecal intubation time for all steering modules in the first seven sessions. The *table* includes the median (95 % confidence interval) intubation time per session, for each steering module

Experts performed cecal intubation using the conventional angulation wheels in an average of 352 ± 86 s (Table 1; Fig. 4). During these fifteen conventional sessions, two experts performed colonoscopy without severe or extreme pain, once.

**Table 1:** Efficiency, visualization and preference outcomes

	Sessions to achieve end-IT and NP	Sessions to achieve end-IT	Visualization performance (%)	Preference conventional	Preference joystick
Expert (*N* = 5)	8 (±6) (*N* = 3)	6 (±6)	97 (±1) Conventional	2	3
			94 (±5) Joystick		
Novice conventional (*N* = 7)	11 (±6)	5 (±2)	97 (±2)	2	5
Novice joystick (*N* = 7)	12 (±6)	4 (±1)	97 (±2)	3	4

All experts reached their personal intubation time in 6 ± 6 sessions, using the setup with joystick interface. Three experts reached their personal intubation time with no pain score in 8 ± 6 sessions. One expert caused a simulated perforation during his second joystick session and claimed overconfidence in scope insertion. Withdrawal time and visualization performance were not significantly different between experts using the conventional or joystick platform, with *p* = 0.92 and 0.68.

There was no significant difference between the number of sessions needed to reach IT or IT + NP for novices using the conventional or joystick platform, with *p* = 0.32 and 0.81. Withdrawal time and visualization performance were also not significantly different between novices using the conventional and novices using the joystick platform, with *p* = 0.17 and 0.43.

Three experts, five conventional novices and four joystick novices preferred the joystick steering method to guide the endoscope tip. The others preferred the conventional angulation wheels (Table 1).

## Discussion

We developed an add-on platform that allows single-person control of a conventional endoscope and instrument at the intervention site. The aim of this study was to determine if endoscopists can reach the intervention site using this platform. The study shows that both experts in endoscopy and novices are able to complete the most difficult colonoscopy case of a training simulator. Experts are able to learn to work with the platform in a relatively short training period. Furthermore, novices performed colonoscopy tasks equally well compared to using the conventional angulation wheels.

Reaching the intervention site in the torturous and flexible large bowel requires a complex combination of endoscope manipulation techniques. Despite intuitive and ergonomic shortcomings of the conventional endoscope, experts are competent in scope manipulation without causing excessive patient pain [8, 20]. Previous studies showed that remote actuation platforms could not yet compete with the efficiency of conventional endoscope control [21–23]. The setup of these platforms prevented adequate scope manipulation [23–25]. This study shows that our setup and interface enable at least as efficient manipulation of the endoscope and effective visualization of the bowel wall.

Not all experts were able to finish their learning curve with no severe or extreme pain scores. Also few conventional sessions were without severe or extreme pain scores. In hindsight, the NP endpoint may have been too strict, making it too difficult to reach the learning curve’s endpoint. There are alternatives, such as requiring that more than 97 % of the procedure time is free of patient discomfort, used by Ahlberg et al. [26]. However, this was considered too easy for this task and would fail to enforce realistic loop detection and straightening techniques. A combination of no extreme pain and a 97–98 % of discomfort free procedure time could be a solution for next studies.

We asked the novices to practice until reaching the expert’s average intubation time plus one standard deviation. This can be considered a high training standard. Nevertheless, the average expert intubation time was with 438 s close to the 7 min on the same simulator case that trainees needed before starting clinical colonoscopies in the training study by Ahlberg et al. [26]. Also, since all novices reached the endpoints, they were confirmed not too challenging.

Both conventional and joystick groups showed a large spread (50 % of the average) in the number of sessions that were required to reach the endpoints. We consider this spread to be the reflection of differences in personal physical and cognitive skills of the inexperienced participants. Since the spread was equally divided between the conventional and joystick groups, it is not attributed to either steering method.

The platform received a low preference rate to use as a tool to navigate an endoscope through the colon. The main reason is the lack of haptic feedback from the tension on the angulation wheels. The addition of a motor drive unit intercepts this haptic signal. Instead we inform users with a visual tip bending diagram. A similar compromise was seen in robotic laparoscopic surgery, which also lacks haptic feedback of instruments. Considering the research carried on about haptic feedback, we expect that a workable solution will be available in the future.

This study indicates that the add-on platform with joystick interface has the potential to guide a flexible endoscope to intervention sites throughout the colon. We will continue our work on the original goal of the device: performing complex therapeutic interventions.

## References

[1] Williams JE, Le TD, Faigel DO (2011). Polypectomy rate as a quality measure for colonoscopy. Gastrointest Endosc.

[2] Jiang M, Joseph L (2012). Different screening definitions have little impact on polypectomy rate estimates. Can J Gastroenterol.

[3] Tanaka S, Oka S, Kaneko I, Hirata M, Mouri R, Kanao H, Yoshida S, Chayama K (2007). Endoscopic submucosal dissection for colorectal neoplasia: possibility of standardization. Gastrointest Endosc.

[4] ASGE/SAGES (2006). ASGE/SAGES working group on natural orifice translumenal endoscopic surgery white paper October 2005. Gastrointest Endosc.

[5] Reavis K, Melvin W (2008). Advanced endoscopic technologies. Surg Endosc.

[6] Teoh AYB (2010). Current developments in natural orifices transluminal endoscopic surgery: an evidence-based review. World J Gastroenterol.

[7] Shah SG, Saunders BP, Brooker JC, Williams CB (2000). Magnetic imaging of colonoscopy: an audit of looping, accuracy and ancillary maneuvers. Gastrointest Endosc.

[8] Sedlack RE (2011). Training to competency in colonoscopy: assessing and defining competency standards. Gastrointest Endosc.

[9] Yamamoto H, Mönkemüller K, Wilcox C, Muñoz-Navas M (2010). Endoscopic submucosal dissection for colorectal tumors. Interventional and therapeutic gastrointestinal endoscopy.

[10] Berr F, Ponchon T, Neureiter D, Kiesslich T, Haringsma J, Kaehler GF, Schmoll F, Messmann H, Yahagi N, Oyama T (2011). Experimental endoscopic submucosal dissection training in a porcine model: learning experience of skilled Western endoscopists. Dig Endosc.

[11] Oka S, Tanaka S, Kaneko I, Kanao H, Chayama K (2007). Techniques and pitfalls of endoscopic submucosal dissection for colorectal tumors. Dig Endosc.

[12] Rösch T, Adler A, Pohl H, Wettschureck E, Koch M, Wiedenmann B, Hoepffner N (2008). A motor-driven single-use colonoscope controlled with a hand-held device: a feasibility study in volunteers. Gastrointest Endosc.

[13] Cosentino F, Tumino E, Passoni GR, Morandi E, Capria A (2009). Functional evaluation of the endotics system, a new disposable self-propelled robotic colonoscope: in vitro tests and clinical trial. Int J Artif Organs.

[14] Vucelic B, Rex D, Pulanic R, Pfefer J, Hrstic I, Levin B, Halpern Z, Arber N (2006). The aer-o-scope: proof of concept of a pneumatic, skill-independent, self-propelling, self-navigating colonoscope. Gastroenterology.

[15] Ruiter JG, Bonnema GM, Voort MC, Broeders IAMJ (2013) Robotic control of a traditional flexible endoscope for therapy. J Robot Surg (**In press**) 10.1007/s11701-013-0405-4 27000918

[16] Ruiter JG, Rozeboom ED, Van der Voort MC, Bonnema GM, Broeders IA (2012) Design and evaluation of robotic steering of a flexible endoscope. IEEE international conference on biomedical robotics and biomechatronics

[17] Rozeboom E, Ruiter J, Franken M, Broeders I (2014). Intuitive user interfaces increase efficiency in endoscope tip control. Surg Endosc.

[18] Ruiter JG (2013) Robotic flexible endoscope, 1st edn, Enschede

[19] Rex DK, Petrini JL, Baron TH, Chak A, Cohen J, Deal SE, Hoffman B, Jacobson BC, Mergener K, Petersen BT, Safdi MA, Faigel DO, Pike IM (2006). ASGE quality indicators for colonoscopy. Gastrointest Endosc.

[20] Shergill A, McQuaid K, Rempel D (2009). Ergonomics and GI endoscopy. Gastrointest Endosc.

[21] Allemann P, Ott L, Asakuma M, Masson N, Perretta S, Dallemagne B, Coumaros D, De Mathelin M, Soler L, Marescaux J (2009). Joystick interfaces are not suitable for robotized endoscope applied to NOTES. Surg Innov.

[22] Kume K, Kuroki T, Sugihara T, Shinngai M (2011). Development of a novel endoscopic manipulation system: the endoscopic operation robot. World J Gastrointest Endosc.

[23] Eckl R, Gumprecht JJ, Strauss G, Hofer M, Dietz A, Lueth TC (2010) Comparison of manual steering and steering via joystick of a flexible rhino endoscope. 32nd annual international conference of the IEEE engineering in medicine and biology society. IEEE engineering in medicine and biology society, Conference, 2010, vol 2010, pp 1234–1237 10.1109/IEMBS.2010.5626433 21096123

[24] Fang C, Sang W, Gumprecht JDJ, Member S, Strauss G, Lueth TC (2012) Image-guided steering of a motorized hand-held flexible rhino endoscope in ENT diagnoses. 2012 IEEE international conference on robotics and automation, 2012, pp 1086–1091

[25] Reilink R, Kappers AML, Stramigioli S, Misra S (2013). Evaluation of robotically controlled advanced endoscopic instruments. Int J Med Robot Comput Assist Surg.

[26] Ahlberg G, Hultcrantz R, Jaramillo E, Lindblom A, Arvidsson D (2005). virtual reality colonoscopy simulation: a compulsory practice for the future colonoscopist?. Endoscopy.

